# Triple-Band Reconfigurable Monopole Antenna for Long-Range IoT Applications

**DOI:** 10.3390/s23125359

**Published:** 2023-06-06

**Authors:** Muhammad Sani Yahya, Socheatra Soeung, Narinderjit Singh Sawaran Singh, Zainab Yunusa, Francis Emmanuel Chinda, Sharul Kamal Abdul Rahim, Umar Musa, Nursyarizal B. M. Nor, Cheab Sovuthy, Ghulam E. Mustafa Abro

**Affiliations:** 1 Department of Electrical and Electronic Engineering, Universiti Teknologi PETRONAS, Bandar Seri Iskandar 32610, Perak, Malaysia; 2 Department of Electrical and Electronic Engineering, Abubakar Tafawa Balewa University, Bauchi 740272, Nigeria; 3 Faculty of Data Science and Information Technology (FDSIT), INTI International University, Persiaran Perdana BBN, Putra Nilai, Nilai 71800, Negeri Sembilan, Malaysia; narinderjits.sawaran@newinti.edu.my; 4 Department of Electrical Engineering, University of Hafr Al Batin, Hafr Al Batin 39524, Saudi Arabia; 5 Wireless Communication Centre, Universiti Teknologi Malaysia, Skudai 81310, Johor, Malaysia; 6 Faculty of Electrical and Electronic Engineering, University Tun Hussein Onn, Parit Raja 86400, Johor, Malaysia; 7 FILPAL (M) Sdn. Bhd., Bayan Lepas, George Town 11900, Penang, Malaysia

**Keywords:** antenna, energy efficiency, IoT, LoRa, monopole, PIN diode, reconfigurable

## Abstract

In this study, a novel reconfigurable triple-band monopole antenna for LoRa IoT applications is fabricated on an FR-4 substrate. The proposed antenna is designed to function at three distinct LoRa frequency bands: 433 MHz, 868 MHz, and 915 MHz covering the LoRa bands in Europe, America, and Asia. The antenna is reconfigurable by using a PIN diode switching mechanism, which allows for the selection of the desired operating frequency band based on the state of the diodes. The antenna is designed using CST MWS^®^ software 2019 and optimized for maximum gain, good radiation pattern and efficiency. The antenna with a total dimension of 80 mm × 50 mm × 0.6 mm (0.12$\lambda_{0}\times 0.07\lambda_{0}$ × 0.001$\lambda_{0}$ at 433 MHz) has a gain of 2 dBi, 1.9 dBi, and 1.9 dBi at 433 MHz, 868 MHz, and 915 MHz, respectively, with an omnidirectional H-plane radiation pattern and a radiation efficiency above 90% across the three frequency bands. The fabrication and measurement of the antenna have been carried out, and the results of simulation and measurements are compared. The agreement among the simulation and measurement results confirms the design’s accuracy and the antenna’s suitability for LoRa IoT applications, particularly in providing a compact, flexible, and energy efficient communication solution for different LoRa frequency bands.

## 1. Introduction

The Internet of Things (IoT) is a fast-growing network that globally interconnects objects, supporting various input/output devices, actuators, and sensors for real-time data collection, control, analysis, and sharing using standard communication protocols. The IoT has had a great influence on our daily lives and has evolved from machine-to-machine communication to connecting people, objects, data, and services. It is considered a key enabler of Cyberphysical Systems (CPSs), which use IoT to link the physical and virtual worlds [1].

One of the critical challenges in IoT is developing communication protocols that meet the energy efficiency, wide signal coverage, energy conservation, affordability, and prolonged battery duration requirements of IoT devices. Low-Power Wide-Area Network (LPWAN) technology is a solution that meets these requirements and has LoRa as one of its major contenders. LoRa is a patented radio modulation technology that utilizes unlicensed frequency bands below 1 GHz and is suitable for IoT applications because of its availability globally and lower cost. The aim of LoRa is to enable IoT devices to communicate over long ranges with improved network capacity, secured data transmission, reduced device cost, and low power consumption [2,3,4,5,6].

Owing to regulatory requirements, the operating frequency bands for LoRa in the ISM bands differ according to regions and countries. For example, within the band of 410–441 MHz in China, about 32 channels were defined for LoRa applications [7]. In Europe, the designated frequencies for LoRa are 868 MHz and 433 MHz, while in America, the operating frequency is 915 MHz [8]. Also, the operating frequency for LoRa in India is 865 MHz [9].

Robust and sophisticated communication protocols and efficient hardware are vital to accomplishing an efficient communication network. Communication devices use antennas to transmit and receive signals; therefore, an antenna is an integral part of LoRa IoT devices [10,11,12,13,14]. Considering the operating frequencies of LoRa (below 1 GHz) and the ever-increasing need for IoT devices to be small and economically low-cost, it is incredibly challenging to design a miniaturized LoRa antenna that can be integrated alongside compact IoT devices [15].

Conventional LoRa modules are equipped with a single whip antenna operating at a fixed frequency. On the other hand, modern IoT devices communicate over multiple frequency bands. The device will be cumbersome if several single-frequency resonant antennas are used. Hence, there is a need for a single multiband antenna for IoT communication to extend network capacity [16]. It should be noted that wideband and multiband antennas operate at a wide/single frequency band. They cannot be changed to a particular frequency band based on the user’s demand. Instead, they radiate energy in all their designed frequency bands leading to higher energy consumption and interference with other devices. This limitation can hinder the usage of such antennas in emerging IoT devices.

Reconfigurable antennas are a better choice to overcome these challenges. The concept of reconfigurability in antenna design refers to its capability to adjust its characteristics, such as polarization, radiation pattern or resonant frequency. A reconfigurable frequency antenna can switch its operating frequency to a chosen band. Therefore, the frequency spectrum can be efficiently utilized [17,18].

The modern printed circuit technology enables easy manufacture of Microstrip Patch Antennas (MPAs), which are also lightweight and mechanically robust. Moreover, these antennas can be integrated with both planar and non-planar surfaces [19,20,21]. It is, therefore, the best candidate to be used in LoRa IoT communications. Despite these advantages, it remains difficult to design a miniaturized antenna for LoRa IoT applications at frequencies below 1 GHz.

There have been a number of documented antenna designs [7,16,22,23,24,25,26,27,28,29,30,31,32,33,34,35,36,37,38,39,40,41,42] for LoRa applications over the last decade. The authors in [7] reported the design of a miniaturized antenna for LoRa sensor node IoT application on an FR-4 substrate. The antenna employs a Planar Inverted Antenna (PIFA) structure with additional resonance created by cutting slots on the ground plane. This results in a downward shift of the PIFA’s resonance frequency from 450 MHz to 410 MHz. The antenna was designed to fit at the edge of the LoRa sensor node circuit board, covering an area of 125 mm × 20 mm × 1.6 mm. It achieved a realized gain greater than −6 dBi and a Voltage Standing Wave Ratio (VSWR) of less than 3.

In [16], a miniaturized dual-band antenna prototyped on an FR-4 material for LPWAN application was reported. The antenna operates at 433 MHz and 868 MHz and has an overall size of 90 mm × 30 mm × 1.6 mm. It used the geometry of the logo for the Université Cote d’Azur to achieve a maximum gain of −4.1 dB at 433 MHz and −2.2 dB at 868 MHz. However, the antenna is non-reconfigurable, and its gain is insufficient for long-range communication.

In [22], an FR-4 material was employed to develop a rectangular MPA for LoRaWAN application at 433 MHz. Cutting two (2) T-Shaped slots on its ground plane enhanced the antenna’s gain and efficiency. It has accomplished a gain of 2.194 dB with a total dimension of 210.82 mm × 164.79 mm × 5.5 mm. A novel LoRa antenna operating at 868 MHz for overwater and underwater surface communication was reported in [23]. With a footprint of 120 mm × 70 mm × 2.4 mm, the antenna has achieved a gain of 2.11 dB. An E-shaped dual-band energy harvesting antenna designed to operate in EGSM-900 and LoRaWAN mobile communication networks was presented in [28]. The antenna design was carried out on an FR-4 substrate and optimized to a total size of 102 × 81 × 1.6 mm^3^ using HFSS and Grey Wolf Optimizer (GWO).

An IoT terminal with a dimension of 300 × 30 × 0.8 mm^3^ designed to support three antennas operating at four frequency bands was presented in [33]. The terminal comprises: dual band GSS antenna at 1.57 GHz and 1.21 GHz for the Galileo L1 and L2 frequencies, respectively, 2.4 GHz LoRa antenna, and 868 MHz LoRa antenna. The realized maximum gain of the GSS antenna was 3.05 dBi for both L1 and L2 frequencies, 4.17 dBi at 2.4 GHz and 3.36 dBi at 868 MHz. Each of these antennas operates in one LoRa frequency and has a large footprint; hence, they are not suitable for compact IoT devices.

A reconfigurable pattern antenna at 868 MHz was reported in [41]. The antenna has a compact dimension of 80 × 55 mm^2^ and is switched electronically to achieve four patterns. With a single-slot radiator, the antenna achieved a peak gain greater than 0.5 dB, whereas a gain of 1.6 dB was achieved in the monopole configuration. This antenna has compact and reconfigurable characteristics but is fixed to only one LoRa frequency.

A compact wideband double leaf-shaped MPA was reported in [42]. The antenna was prototyped on an FR-4 material and had a total size of 22 × 34 mm^2^. It has covered all the LoRa bands in the sub-1 GHz: 915 MHz, 868 MHz, and 433 MHz, with a peak gain of 2.56 dB. This antenna is compact but cannot be tuned to suppress or select a particular LoRa frequency band.

This work presents a compact, novel, triple-band frequency reconfigurable monopole antenna for LoRa IoT applications on commercially available and cheaper FR-4 material. The antenna with a dimension of 80 mm × 50 mm × 0.6 mm can be tuned using two (2) RF (Radio Frequency) PIN diodes (D1 and D2) placed strategically in the branches of the antenna. Switching these diodes enables the antenna to operate at 915 MHz, 868 MHz, and 433 MHz, depending on the state of the diodes. These covered the LoRa frequencies across the globe. Meandered radiating monopoles and partial ground plane are employed to achieve compact size and impedance matching of the antenna. To the best of our knowledge, this is the first compact reconfigurable antenna that covered all the LoRa frequencies.

The subsequent sections of this paper have been organized as follows: Section 2 discusses the theory, structure, and reconfiguration mechanisms of the novel triple-band LoRa monopole antenna. Section 3 provides a detailed analysis of the comparison between measurement and simulation results for the antenna. Finally, Section 4 gives the conclusion of the work.

## 2. Design Methodology

The endless demand for compact and low-cost wireless IoT systems has increased the necessity for miniaturized, compact, and portable antennas. Such compact antennas should fit easily into every small space for IoT applications. Here, the proposed antenna’s methodology, basic structure, theory, design, and reconfiguration mechanism are presented.

### 2.1. Theory and Geometry of the Antenna

**Figure 1:**
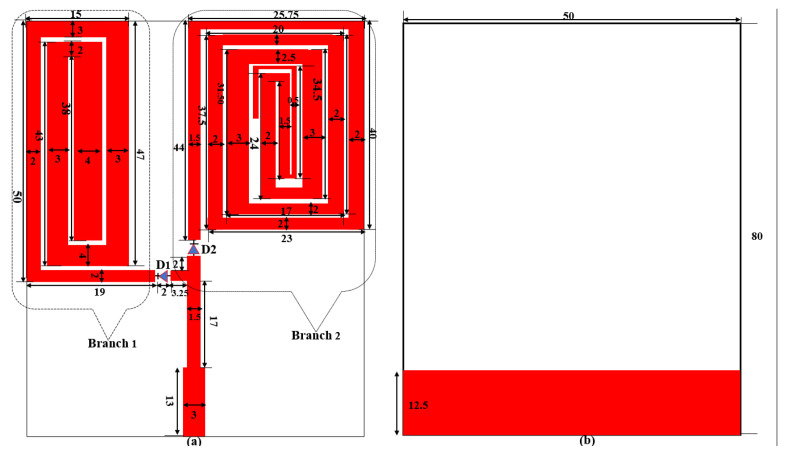
Geometry of the antenna (**a**) Front (**b**) Back.

The geometry of the proposed antenna is shown in Figure 1. It comprises two (2) branches of radiating elements each containing spirals of rectangular meandered monopoles on the top surface of the substrate and a partial copper ground plane on the bottom layer. Each branch has its specific resonance, which is controlled by RF switches. Meandered microstrip lines are considered a powerful miniaturization technique in antenna design. They are used to lengthen the path of current flow on an antenna’s surface to produce lower frequencies resonance. Figure 1a depicts the top view of the triple band LoRa monopole antenna designed on a 0.6 mm thick FR-4 substrate of dimensions 80 mm × 50 mm.

To make the antenna integrable in emerging IoT devices, standard credit card dimensions of 85.6 mm × 53.98 mm are targeted to be the size of the proposed antenna. The type and characteristics of the substrate were chosen to make the antenna planar, cheaper, and easy to fabricate. The substrate has $\varepsilon_{r}$ (relative permittivity) of 4.4 and tan δ (loss tangent) of 0.02. To improve the antenna’s performance in terms of gain and efficiency, a partial copper ground plane (35 m) is used as shown in Figure 1b. A Microstrip line with a dimension of 3 mm is used to excite the antenna to achieve 50 Ω impedance matching. Two slots of width 2 mm each are cut at appropriate positions on the branch 1 and branch 2 so that RF switches represented by D1 and D2 can be integrated into the antenna for switching among LoRa frequencies. The length (L) of the spiral meandered monopoles in each branch that will produce resonance at a specific frequency is determined using the Equations (1) and (2) as presented in [43,44]. A monopole antenna is theoretically a quarter wavelength antenna having its radiating elements and ground plane equal to 1/4th of the wavelength of the resonance frequency of the antenna. This concept is used to fold the monopoles into a spiral pattern to produce the required resonance at 433 MHz, 868 MHz, and 915 MHz. Then, PIN Diodes are used to select among these frequencies depending on the state of the diodes. The design, simulation, optimization, and antenna analysis are carried out in CST MWS^®^ software environment. The detailed dimensions of the antenna parameters in millimetres are presented in Figure 1. In the simulation, the antenna is excited using a waveguide port. The efficiency of an antenna increases when it is excited at the correct position. This also reduces the reflection coefficient of the antenna ($S_{11}$).

(1) $$L=\frac{\lambda }{4},$$

where λ is the guided wavelength at each resonant frequency $\left(f_{r}\right)$ and it is given by:

(2) $$\lambda_{f_{r}}=\frac{c}{f_{r}\sqrt{\varepsilon_{e}}},$$

*c* = speed of light in vacuum and $\varepsilon_{e}$ is the effective permittivity

By folding the monopole conductors back and forth to form a meandered-line antenna as shown in branches 1 and 2 of Figure 1, a compact antenna is realised which is capable of operating at three distinct frequencies, 915 MHz, 868 MHz, and 433 MHz, depending on the status of the switching elements D1 and D2.

### 2.2. Frequency Switching Techniques

**Figure 2:**
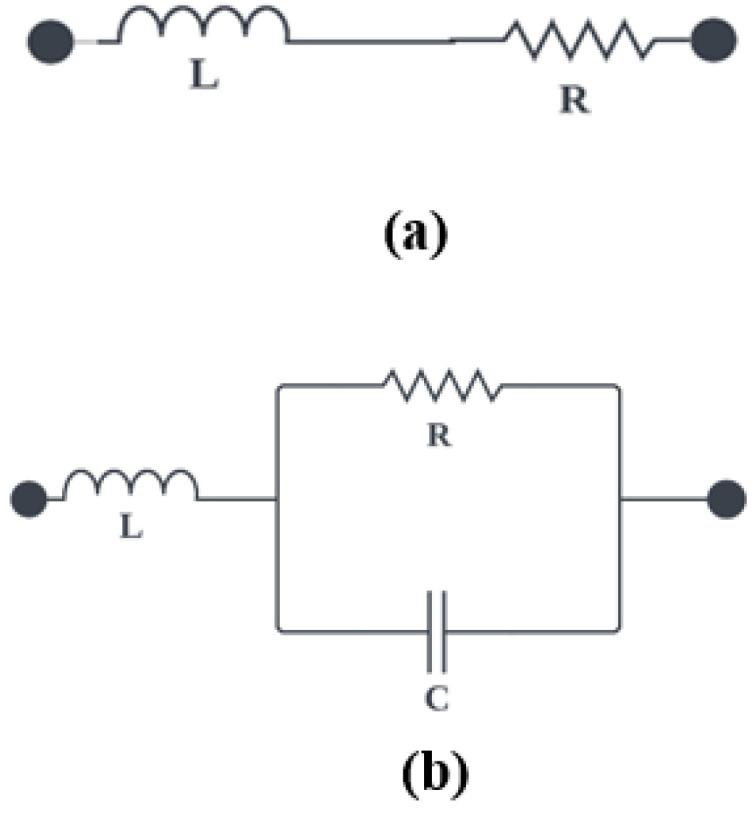
Equivalent circuit (**a**) ON state (**b**) OFF state.

Frequency reconfigurability in an antenna refers to the ability to alter the path that current follows on the antenna’s surface, thereby allowing for a shift in resonance to the desired frequency band. Instead of using multiple antennas operating at different frequencies, a reconfigurable frequency antenna can achieve the same function and simultaneously reduce cost and save space. Various techniques and methods are used to achieve frequency reconfiguration, including switches or slots on an antenna’s radiating elements. Electronic switches used for reconfiguration in antenna include Field-Effect-Transistors (FET), Varactor diodes, Micro-Electromechanical Systems (MEMS), PIN diodes, etc. PIN diodes have received much attention for use as a switch in antenna due to their low cost, moderate isolation, less complicated biasing circuitry, and ease of integration with the antenna elements [45,46]. In this work, two PIN diodes D1 and D2 (BAR50-02V) from Infineon, are used to switch the antenna to operate at different LoRa frequencies. BAR50-02V is a silicon PIN diode with low forward resistance and extremely low harmonics distortions. It can operate from >10 MHz to 6 GHz. When both the diodes are ON, current flows from the feed to branch 1 and branch 2 of the antenna to produce an effective resonance at 915 MHz. When D1 is ON, and D2 is OFF, a resonance at 433 MHz is produced. The antenna operates at 868 MHz when D2 is ON, and D1 is OFF. The schematic for the equivalent circuits of the ON and OFF states of PIN diode are shown in Figure 2.

**Figure 3:**
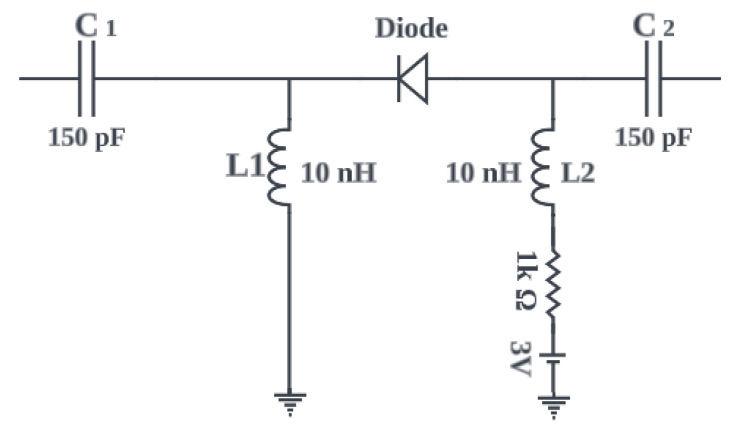
Biasing circuit of PIN Diode.

It is evident from Figure 2 that, in the ON state, the PIN diode is represented by a series combination of a resistor (R) and an inductor (L). While in the OFF state, it is represented by an inductor (L) in series with a parallel combination of a resistor (R) and a capacitor (C), as shown in Figure 2b. The values of these parameters for the ON and OFF states are obtained from the diodes’ technical data sheets. In the ON state, the values of R and L are small such that they behave as a short circuit to allow current to flow through the radiating loops. While in the OFF state, the values are selected such that the circuit blocks the current flow through the radiating loops. In the simulation, the ON state of the PIN diode is modelled using resistor of 1 Ω without taking the effects of the capacitor and inductor because in both the PIN diode model and the RLC lumped model, the ON-state of diode behaves as a short circuit to allow the flow of current to the radiating monopoles. In contrast, 20k Ω resistance is used to model the OFF state of the diode to block the flow of current to the radiating monopoles. Alternatively, touchstone files are used in CST to provide the S-Parameters of the diodes at ON and OFF states. This is considered the best method to get the response of the diodes at ON and OFF states for different ranges of frequencies. It is observed that the diodes behaved in a similar way using the two techniques. In the prototype antenna, the two PIN diodes are soldered appropriately on branch 1 and branch 2. The biasing voltage of the PIN diode is provided by 2-AAA batteries through jumper wires. Figure 3 depicts the biasing circuit of the PIN diode.

## 3. Results and Analysis

**Figure 4:**
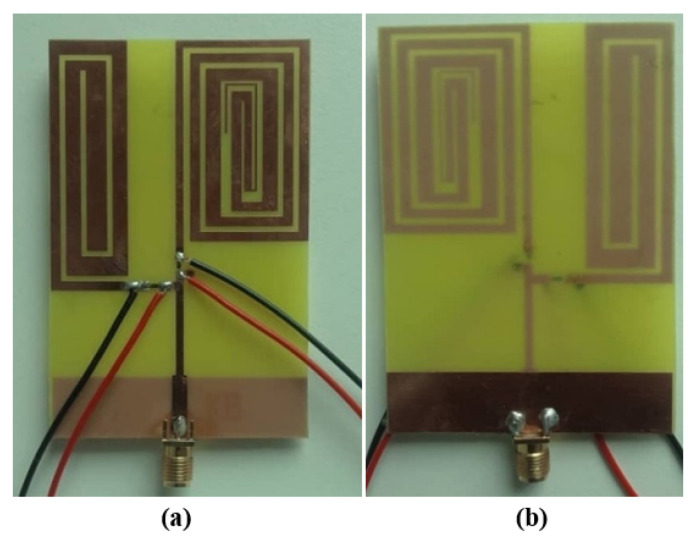
Prototype antenna (**a**) Front (**b**) Back.

This section presents a discussion and analysis of the performance of the proposed antenna, with a focus on the return loss ($S_{11}$), distribution of surface currents, radiation patterns, gain, efficiency, etc. The results of simulations and measurements are compared in terms of these metrics. The fabricated antenna depicted in Figure 4 is fabricated on a cheaper substrate material (FR-4). The antenna’s $S_{11}$, gain, and radiation pattern were measured using VNA (Vector Network Analyzer) and an anechoic chamber.

### 3.1. Return Loss $S_{11}$

**Figure 5:**
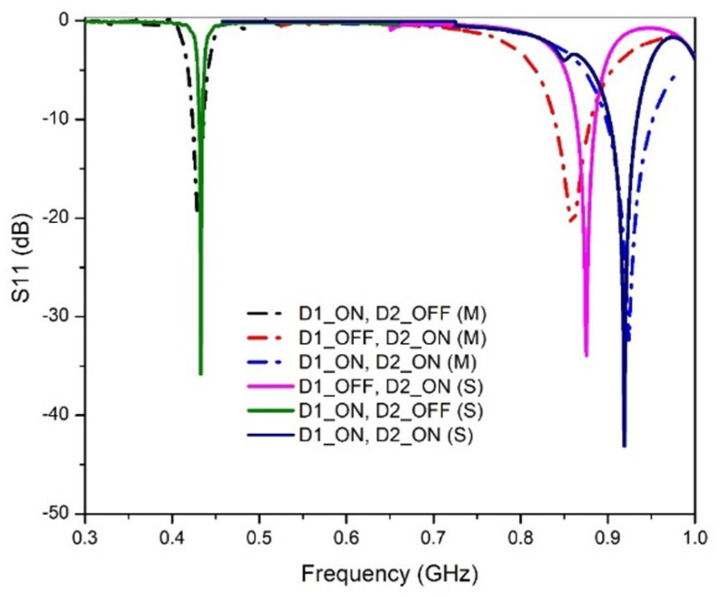
Comparison of $S_{11}$ of the proposed antenna at different states of the diodes.

The simulated and measured $S_{11}$ of the antenna at different switching conditions of the PIN diodes are shown in Figure 5. It is observed that, depending on the diode’s state, the antenna operates at 433 MHz, 868 MHz, and 915 MHz. When D1 is ON and D2 is OFF, the antenna operates at 433 MHz with a magnitude of −35.8 dB and −19.9 dB for simulation and measurement, respectively. This is due to the fact that D2 is reversed biased; hence, current cannot flow through it to branch 2. During the OFF state of D1 and ON state of D2, the antenna operates at 868 MHz with a magnitude of −33.9 dB and −18.1 dB for simulation and measurements, respectively. This is due to the isolation of branch 1 as a result of D1 being in reverse bias. The proposed antenna operates at 915 MHz when both D1 and D2 are in the ON states. The current from the feed flows freely to branch 1 and branch 2, which results in a combined effect of producing a single band at 915 MHz. The magnitude of the return loss at 915 MHz is −45.9 dB and −18.9 dB for simulation and measurements, respectively. The summary of the functionality of the antenna at different states of the diode is presented in Table 1. In each case, the simulated and measured results agreed with each other. The proposed antenna covers all the LoRa bands (433 MHz, 868 MHz, and 915 MHz), making it the first single-solution reconfigurable antenna with these characteristics. The antenna is well-matched in each case with very small return loss values.

#### 3.1.1. Parametric Studies

In order to optimize the performance of the antenna design, parametric studies were conducted on some key parameters: the length of the feed (L${}_{f}$) and its width (W${}_{f}$) as well as the length of the ground plane (L${}_{g}$) under different switching states of D1 and D2. The purpose of these studies is to identify the values of L${}_{f}$, W${}_{f}$, and L${}_{g}$ that would yield lower return loss and good matching at the desired frequencies of operation.

For the investigation of L${}_{f}$, different lengths are considered: 13 mm, 20 mm, and 26 mm. The return loss and matching characteristics are analyzed for each length. The results indicated that the optimum length that provided the desired performance is 13 mm. At this length, the antenna exhibited lower return loss and achieved good matching.

Similarly, the study on the W${}_{f}$ is conducted using different widths: 3 mm, 4 mm, 5 mm, and 6 mm. The goal is to determine the width that would lead to improved performance in terms of return loss and matching. The analysis revealed that a width of 3 mm yielded good matching, and the antenna exhibited lower return loss at this width.

It is worth noting that as the L${}_{f}$ increased, the matching deteriorated, indicating a diminishing performance as the length deviated from the optimum value of 13 mm. Similarly, the W${}_{f}$ showed a similar trend, with an increase in width, resulting in a degradation of matching.

**Figure 6:**
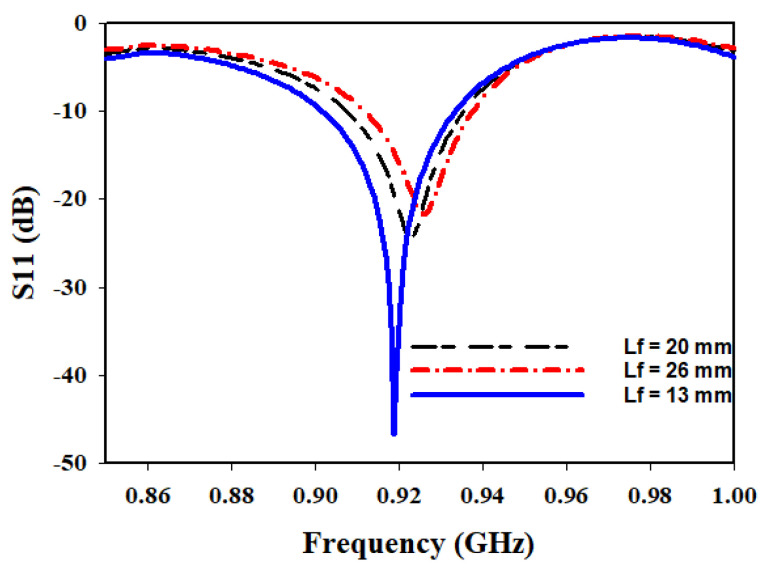
Variation of L${}_{f}$.

Figure 6 presents the variation of the L${}_{f}$ at 915 MHz, displaying the corresponding $S_{11}$ and matching characteristics for the different lengths considered in the parametric study. The plot clearly illustrates that the performance is significantly improved when the feed length is set to 13 mm.

**Figure 7:**
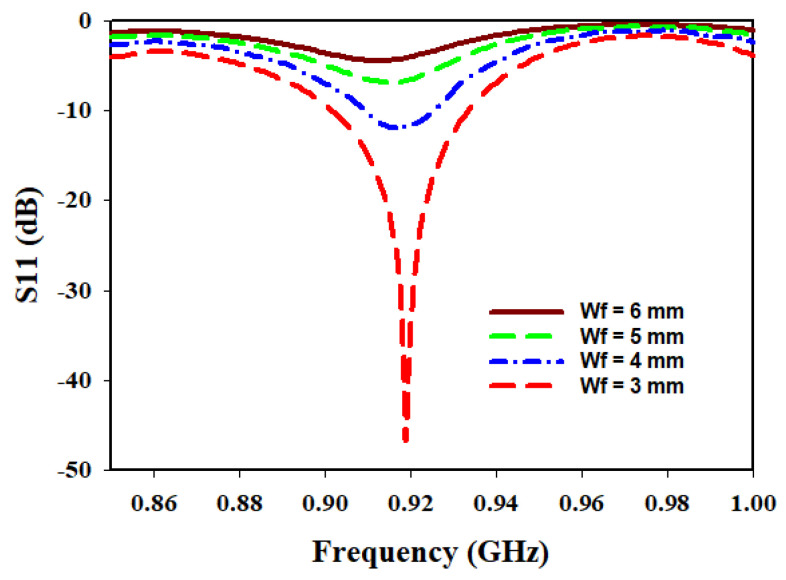
Variation of W${}_{f}$.

Figure 7 showcases the variation of W${}_{f}$ at 915 MHz, demonstrating the associated return loss and matching characteristics for the different widths examined. The plot demonstrates that a width of 3 mm offers superior matching compared to the other widths tested, reinforcing the importance of selecting an appropriate width for achieving desired performance.

These parametric studies play a crucial role in identifying the optimal values of the feed length and width, enabling the design of the antenna with lower return loss and improved matching at all the desired operating frequencies.

**Figure 8:**
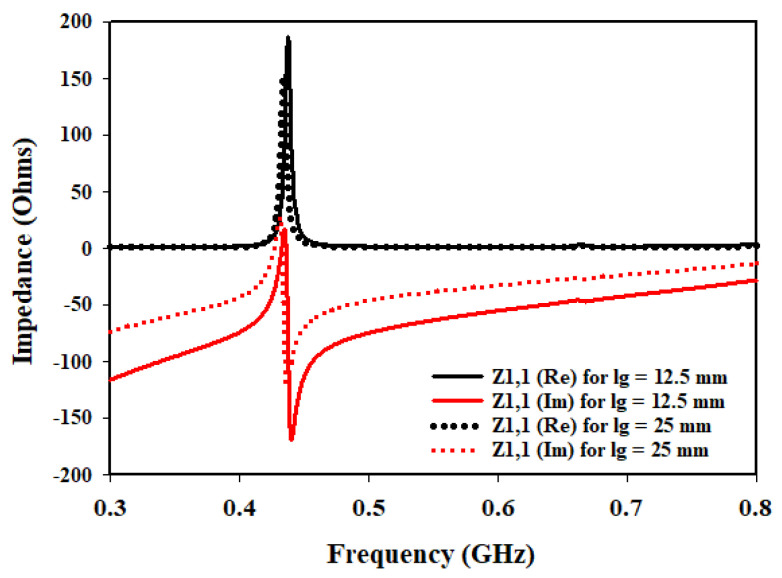
Effects of variation of L${}_{g}$ on input impedance at 433 MHz.

**Figure 9:**
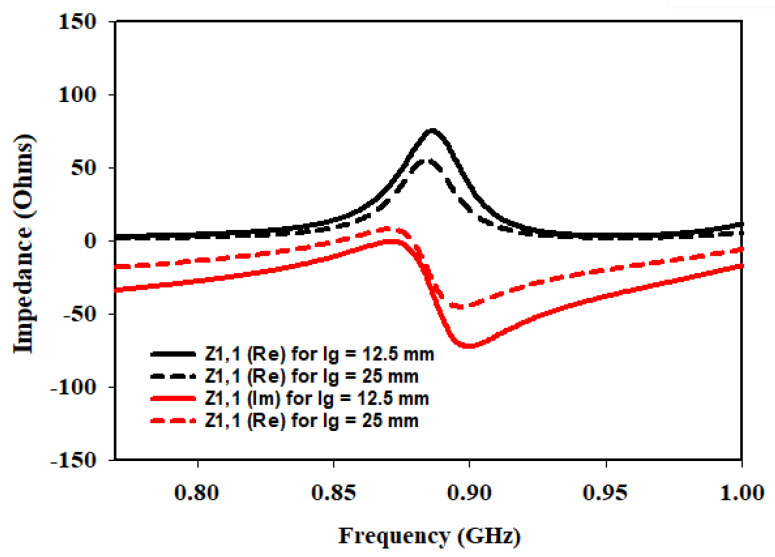
Effects of variation of L${}_{g}$ on input impedance at 868 MHz.

**Figure 10:**
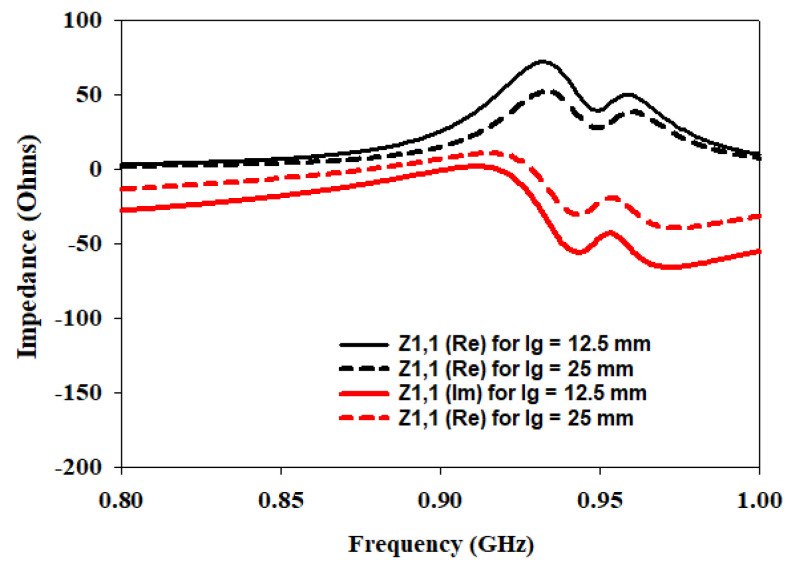
Effects of variation of L${}_{g}$ on input impedance at 915 MHz.

To further enhance the understanding and optimization of the antenna design, an additional parameter is investigated: the length of the ground plane L${}_{g}$. The performance of the antenna is studied at different switching states of the diodes while considering two ground plane lengths: 12.5 mm and 25 mm. The analysis focused on the input impedance at three different frequencies: 433 MHz, 868 MHz, and 915 MHz. Figure 8, Figure 9 and Figure 10 present the corresponding plots of Z1,1 (Re) and Z1,1 (Im) at 433 MHz, 868 MHz and 915 MHz.

In the investigation of the L${}_{g}$, it is observed that when the ground plane is set to 12.5 mm, the antenna exhibits favorable characteristics in terms of input impedance at the specified frequencies. Specifically, at each frequency, Z1,1 (Re) is found to be closer to the desired value of 50 Ω indicating good matching. Additionally, Z1,1 (Im) is also closer to zero, suggesting a better impedance balance.

However, as the L${}_{g}$ is doubled to 25 mm, the input impedance deviated significantly from the desired 50 Ω target. The values of Z1,1 (Re) and Z1,1 (Im) moved further away from the desired values, indicating a deterioration in matching and impedance balance.

These findings highlight the crucial role played by the L${}_{g}$ in achieving optimal antenna performance. When the ground plane length is set to 12.5 mm, the antenna demonstrated improved matching and impedance characteristics at the specified frequencies of 433 MHz, 868 MHz, and 915 MHz. This suggests that a shorter ground plane length helps in achieving a closer match to the desired impedance of 50 Ω, resulting in improved performance.

On the other hand, doubling the ground plane length to 25 mm led to a significant deviation from the desired impedance values. This indicates that an increase in the ground plane length adversely affects the matching and impedance balance, resulting in degraded performance.

The parametric studies involving the L${}_{g}$, in addition to the L${}_{f}$ and W${}_{f}$, have provided valuable insights into optimizing the antenna design. The results emphasize the importance of selecting appropriate values for these parameters to achieve lower return loss, improved matching, and closer adherence to the desired input impedance of 50 Ω at the specified frequencies.

#### 3.1.2. Distribution of Surface Current

The performance of any antenna is greatly influenced by the distribution of surface current on its radiating elements. The surface current distribution on an antenna is crucial in determining its radiation pattern, gain, and directivity. By carefully controlling the current distribution on the radiating elements, the performance of an antenna can be optimized to resonate at specific frequencies for specific applications.

**Figure 11:**
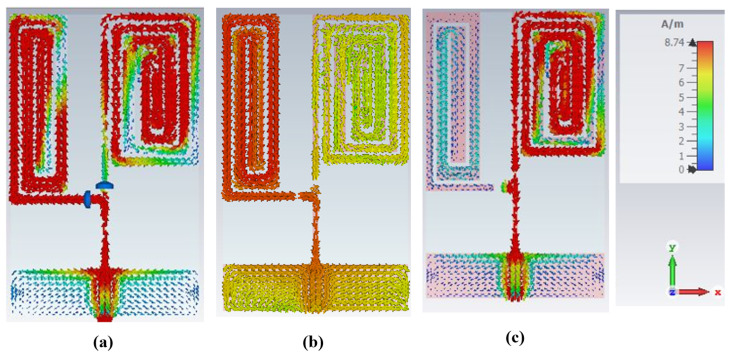
Surface current (**a**) D1: ON, D2: ON (**b**) D1: ON, D2: OFF (**c**) D1: OFF, D2: ON.

Here, the monopole elements on which the surface current density is maximum and responsible for producing resonance at a particular frequency are studied. The surface current distributions at distinct switching states of the diodes are illustrated in Figure 11. It is apparent from Figure 11a that all the elements in branch 1 and branch 2 produced the resonance at 915 MHz when both D1 and D2 are ON. This is due to the free flow of current to both the branches due to D1 and D2 being in a forward bias states. In contrast, when D1 is ON, and D2 is OFF, current does not flow to branch 2; consequently, only the elements of branch 1 radiate and produce a resonance at 433 MHz (Figure 11b). Additionally, when D1 is OFF, and D2 is ON, current does not flow to branch 1 because D1 is reversed-biased; hence, only the elements in branch 2 radiate and generate the resonance at 868 MHz (Figure 11c). In each case, the maximum current density is illustrated by the red colour, as can be visualized from the current density colour ramp.

#### 3.1.3. Radiation Pattern, Gain and Efficiency

**Figure 12:**
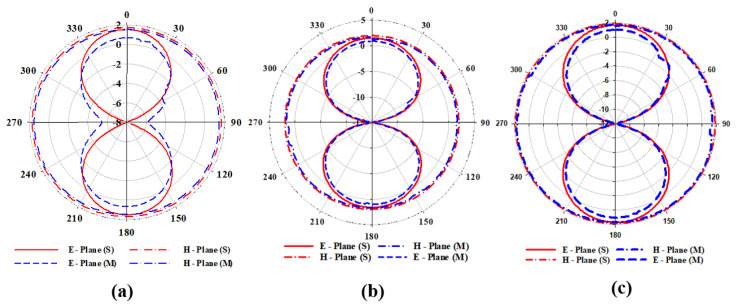
Comparison of radiation patterns of the proposed antenna (**a**) D1: ON, D2: OFF (**b**) D1: OFF, D2: ON (**c**) D1: ON, D2: ON.

**Figure 13:**
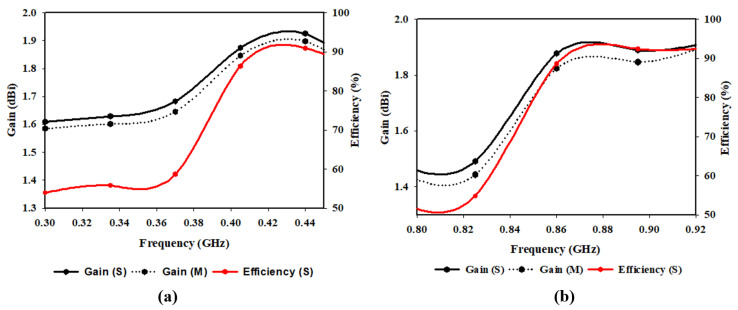
Comparison of gain and efficiency of the proposed antenna (**a**) 433 MHz (**b**) 868 MHz and 915 MHz.

The antenna’s radiation pattern represents how the antenna radiates electromagnetic energy into space. It provides information about the antenna’s directivity, which measures how well it concentrates its energy in a particular direction. It also provides information about the antenna’s gain, which measures the amount of power radiated by the antenna in a specific direction compared to an isotropic radiator. The proposed antenna’s radiation pattern in E and H planes for simulation and measurements at 915 MHz, 868 MHz, and 433 MHz are shown in Figure 12. In Figure 12a, the antenna operates at 433 MHz (D1: ON, D2: OFF) with a peak directivity of 2 dBi. Figure 12b illustrates how the proposed antenna emits radio waves at 868 MHz (D1: OFF, D2: ON) with a peak gain of 1.9 dBi. In Figure 12c, the proposed antenna operates at 915 MHz when both diodes are ON with a gain of 1.9 dBi. In each case, for both simulation and measurement, the proposed antenna has an omnidirectional pattern in H-plane. While the radiation pattern in E-plane is bidirectional, as evidenced by the ’Eight’ shape of the E-plane patterns with a null lobe at θ = 90° for each state of the diode. A bidirectional radiation pattern is often used in wireless communication systems, particularly in wireless sensor networks where the sensor node needs to communicate with two or more other nodes for IoT applications. The simulated and measured results in both planes (E and H) agree. The plots of gain and efficiency of the proposed antenna against frequency are shown in Figure 13. It can be seen that the antenna has almost uniform gain (1.9 dBi) in all its bands of operation with a maximum radiation efficiency of 93% at 915 MHz. This implied that the antenna could transmit and receive signals with relatively equal strength across all its three bands of operation.

A comparison of the performance of the proposed antenna with the existing literature is presented in Table 2. The comparison table provides a comprehensive evaluation of various antennas designed for LoRa applications. From the table, it is evident that the proposed work, which is a monopole antenna, offers superior performance compared to the other works listed.

One of the key advantages of the proposed antenna is its multiband coverage, which spans across three frequency bands: 433 MHz, 868 MHz, and 915 MHz. This makes the antenna suitable for various applications where a multiband antenna is required. Additionally, the proposed antenna has a higher gain of 2 dBi at 433 MHz, which is desirable for long-range communication and signal reception in low signal strength environments.

Another significant advantage of the proposed antenna is its reconfigurability, which allows it to change its operating frequency. This feature enables the antenna to adapt to different communication protocols and environments, improving its performance significantly. Furthermore, the antenna’s compact size of 80 mm × 50 mm × 0.6 mm^3^ makes it suitable for integration into small devices. The monopole technique used in the proposed antenna is well-established and widely used in various applications. This technique is known for its simplicity and ease of design, which makes it attractive for antenna design in various applications.

## 4. Conclusions

In this paper, a novel compact (80 × 50 × 0.6 mm^3^), triple band reconfigurable monopole antenna for LoRa IoT applications was presented. By utilizing two PIN diodes (D1 and D2) to switch the antenna to different modes, resonance at 433 MHz, 868 MHz, and 915 MHz was achieved, depending on the states of the diodes. When D1 is turned ON, and D2 is turned OFF, the antenna operates in the 433 MHz band, which is the designated frequency for LoRa in Europe. Conversely, when D1 is turned OFF, and D2 is turned ON, the antenna operates in the 868 MHz band, which is also used in Europe for LoRa applications. When both diodes are turned ON, the antenna operates in the 915 MHz band, the designated frequency band for LoRa in North America and some Asian countries. To keep the cost of the antenna low for LoRa IoT applications, the substrate and reconfiguration mechanisms chosen were cost-effective. Additionally, the antenna was miniaturized to have a dimension smaller than the size of a standard “credit card”. With a peak gain of 2 dBi and a radiation efficiency above 90%, this proposed antenna is novel in terms of size and performance for LoRa IoT applications. The antenna was prototyped and tested to validate its performance, and the results of measurements are in good agreement with those of simulation.

## Figures and Tables

**Table 1:** Summary of the switching states of the diodes.

Diode	D1	D2	Frequency (MHz)	$S_{11}$ (dB) Simulation	Measurement
State	ON	ON	915	−45.9	−18.9
	ON	OFF	433	−35.8	−19.9
	OFF	ON	868	−33.9	−18.1

**Table 2:** Comparison of the performance of the proposed antenna with available literatures.

Ref.	Year	Bands	Frequency	Substrate	Conductor	Gain	Reconfi-	All LoRa	Size
			(MHz)	Material	Material	(dBi)	Gurable?	Frequencies?	(mm^2^)
[7]	2018	1	402.4–441.6	FR-4 (NA *)	Copper	−6	NO	YES	125 × 20
[23]	2021	1	868	FR-4 (4.3)	Copper	2.11	NO	YES	120 × 70
[25]	2020	1	871	FR-4 (4.4)	Copper	0.58	NO	YES	67.7 × 55
[27]	2019	1	848–950	FR-4 (4.4)	Copper	2.1	NO	YES	40 × 26
[29]	2019	2	868	FR-4 (4.4)	Copper	1.92	NO	NO	100 × 40
			2400			4.2			
[30]	2021	2	400	FR-4 (NA)	Copper	−5	NO	YES	160 × 170
			900.2						
[34]	2021	3	401	FR-4 (4.7)	Copper	−8.5	NO	YES	78 × 88
			466			−5.2			
			868			−2.8			
[41]	2021	1	868	FR-4 (4.4)	Copper	1.6	YES	YES	80 × 55
[42]	2022	Wide band	151.9–1080	FR-4 (4.4)	Copper	2.56	NO	YES	22 × 34
**This work**	**2023**	**3**	**433**	**FR-4 (4.4)**	**Copper**	**2**	**YES**	**YES**	**80 × 50**
			**868**			**1.9**			
			**915**			**1.9**			

* NA = No information available.

## Data Availability

Not applicable.
